# The widespread use of topical antimicrobials enriches for resistance in *Staphylococcus aureus* isolated from patients with atopic dermatitis†

**DOI:** 10.1111/bjd.16722

**Published:** 2018-07-24

**Authors:** C.P. Harkins, M.A. McAleer, D. Bennett, M. McHugh, O.M. Fleury, K.A. Pettigrew, K. Oravcová, J. Parkhill, C.M. Proby, R.S. Dawe, J.A. Geoghegan, A.D. Irvine, M.T.G. Holden

**Affiliations:** ^1^ School of Medicine University of St Andrews St Andrews KY11 9TF U.K.; ^2^ Department of Dermatology Ninewells Hospital Dundee DD1 9SY U.K.; ^3^ School of Medicine University of Dundee Dundee DD1 9SY U.K.; ^4^ National Children's Research Centre Trinity College Dublin Dublin Ireland; ^5^ Paediatric Dermatology Our Lady's Children's Hospital Crumlin, Dublin 12 Ireland; ^6^ Clinical Medicine Trinity College Dublin Dublin Ireland; ^7^ Irish Meningitis and Sepsis Reference Laboratory (IMSRL) Temple Street Children's University Hospital Dublin 1 DO1 YC67 Ireland; ^8^ Department of Medical Microbiology Royal Infirmary of Edinburgh 52 Little France Crescent Edinburgh EH16 4SA U.K.; ^9^ Department of Microbiology Moyne Institute of Preventative Medicine School of Genetics and Microbiology, Trinity College Dublin Dublin 2 Ireland; ^10^ Institute of Biodiversity, Animal Health and Comparative Medicine College of Medical, Veterinary and Life Sciences, University of Glasgow Glasgow G12 8QQ U.K.; ^11^ The Wellcome Sanger Institute Wellcome Trust Genome Campus Hinxton, Cambridge CB10 1SA U.K.

## Abstract

**Background:**

Carriage rates of *Staphylococcus aureus* on affected skin in atopic dermatitis (AD) are approximately 70%. Increasing disease severity during flares and overall disease severity correlate with increased burden of *S. aureus*. Treatment in AD therefore often targets *S. aureus* with topical and systemic antimicrobials.

**Objectives:**

To determine whether antimicrobial sensitivities and genetic determinants of resistance differed in *S. aureus* isolates from the skin of children with AD and healthy child nasal carriers.

**Methods:**

In this case–control study, we compared *S. aureus* isolates from children with AD (*n* = 50) attending a hospital dermatology department against nasal carriage isolates from children without skin disease (*n* = 49) attending a hospital emergency department for noninfective conditions. Using whole genome sequencing we generated a phylogenetic framework for the isolates based on variation in the core genome, then compared antimicrobial resistance phenotypes and genotypes between disease groups.

**Results:**

*Staphylococcus aureus* from cases and controls had on average similar numbers of phenotypic resistances per isolate. Case isolates differed in their resistance patterns, with fusidic acid resistance (Fus^R^) being significantly more frequent in AD (*P* = 0·009). The genetic basis of Fus^R^ also differentiated the populations, with chromosomal mutations in *fusA* predominating in AD (*P* = 0·049). Analysis revealed that Fus^R^ evolved multiple times and via multiple mechanism in the population. Carriage of plasmid‐derived *qac* genes, which have been associated with reduced susceptibility to antiseptics, was eight times more frequent in AD (*P* = 0·016).

**Conclusions:**

The results suggest that strong selective pressure drives the emergence and maintenance of specific resistances in AD.

## Abstract 2

**What's already known about this topic?**

- *Staphylococcus aureus* frequently colonizes individuals with atopic dermatitis (AD), with increasing disease severity correlating with greater bacterial load of the organism.
- Antimicrobial therapies are routinely used in AD for management and prevention of disease flares.

**What does this study add?**

- *Staphylococcus aureus* isolates from children with AD differ in their antimicrobial resistance profiles from those in healthy, nonatopic nasally colonized children.
- Fusidic acid resistance is significantly more prevalent in cases of AD, and arises through distinct genetic mechanisms when compared with healthy controls.
- Carriage of plasmid‐derived genetic determinants associated with antiseptic resistance also clearly differentiated *S. aureus* from cases of AD and controls.

**Linked Editorial:** https://doi.org/10.1111/bjd.17023.

https://doi.org/10.1111/bjd.17167 available online

https://goo.gl/Uqv3dl

Fundamental to the success of *Staphylococcus aureus* as a pathogen has been is its ability to become resistant to almost every class of antibiotic. The sequential introduction of antimicrobials has directly influenced the emergence and spread of the major drug‐resistant lineages of this organism.^1^ Generally we consider the problems posed by resistance in terms of at‐risk populations, for instance methicillin‐resistant *S. aureus* (MRSA) transmission and invasive infection in hospital inpatients. There are specific patient groups who have increased propensity for *S. aureus* carriage and, as a corollary, infection.^2^ Compared with the general population these patients are at higher risk of drug resistance from frequent antimicrobial usage to manage their condition. Patients with inflammatory skin disorders exemplify this.

Atopic dermatitis (AD) is the most common inflammatory skin disease of childhood, affecting up to 25% of children in the U.K.^3^ Individuals with AD are specifically prone to colonization by *S. aureus*. Cumulative observational evidence has shown that 70% of patients with AD carry the bacterium on lesional skin.^4^ Clinically, there is an observable link between increasing disease activity and *S. aureus* carriage. Disease severity correlates with bacterial load^5^ and the immune response mounted against it.^6^ Consequently, antimicrobial interventions form part of routine care in this patient group. There is no uniformly accepted diagnostic definition of colonization vs. infection in AD, and practices pertaining to use of these treatments vary between dermatologists and in the community. Presently, there is a paucity of high‐quality study evidence supporting beneficial outcomes with usage of antimicrobials in the management of AD flares, which raises the issue of whether they should in fact be used at all.^7^, ^8^

We aimed to determine whether there were phenotypic and genotypic differences in antimicrobial resistance patterns in *S. aureus* from the skin of children with AD compared with *S. aureus* asymptomatically nasally carried by children without skin disease.

## Patients and methods

### Ethics

Approval for these studies was obtained from the research ethics committees of Our Lady's Children's Hospital or Temple Street Children's University Hospital, in Dublin, Ireland. Studies were conducted in accordance with the Declaration of Helsinki, and written informed parental consent was obtained.

### Patients

Children aged 0–7 years meeting the U.K. diagnostic criteria for AD^9^ with moderate‐to‐severe disease were recruited through the dermatology clinic at Our Lady's Children's Hospital, between September 2012 and September 2014. Nonatopic, age‐matched controls were recruited during attendance with a noninfectious illness at the emergency department, Temple Street Children's Hospital, during July and August 2009 as part of a separate *S. aureus* nasal carriage study by an independent study team. Full eligibility and exclusion criteria for both studies were exactly as previously described.^10^ Cases were swabbed at a single inflamed skin site, while controls were swabbed from a single nostril, with *S. aureus* isolation proceeding as previously published. All isolates were then subjected to the same analyses. Sample sizes were determined on the basis of what was practical and not from a formal sample‐size requirement estimate for this study.

### Whole genome sequencing

Bacterial DNA extraction was carried out as described previously.^11^ DNA libraries were prepared with a Nextera XT Library Preparation Kit (Illumina, San Diego, CA, U.S.A.) and quantified with an Agilent Bioanalyser (Agilent, Santa Clara, CA, U.S.A.). Libraries were normalized, pooled and sequenced as 250‐bp paired‐end reads with a MiSeq sequencer (Illumina). The sequence data have been deposited in the European Nucleotide Archive under project accession PRJEB25052.

### Bioinformatic analysis

Multilocus sequence types were determined from sequence reads using SRST2.^12^ Single‐nucleotide polymorphisms (SNPs) were identified by mapping sequence reads to the *S. aureus* reference genome MSSA476^13^ using SMALT.^14^ A maximum likelihood phylogeny was constructed using core genome SNPs as described.^11^ Isolate resistance profiles were predicted *in silico* from sequence reads with SRST2 by comparison with previously compiled resistance determinant databases for 18 antimicrobials.^15^, ^16^ Core chromosomal SNPs conferring resistance were identified by manual inspection of the mapping data.

### Antimicrobial sensitivity testing

Antimicrobial sensitivity (AMS) testing was performed on the VITEK 2 instrument (BioMérieux, Marcy‐l’Étoile, France) using AST‐P634 cards following the manufacturer's instructions. Susceptibilities to all major antibiotic classes were tested using minimum inhibitory concentration values determined to benzylpenicillin, oxacillin, erythromycin, clindamycin, tetracycline, fusidic acid, gentamicin, ciprofloxacin, trimethoprim, mupirocin, linezolid, daptomycin, teicoplanin, vancomycin, chloramphenicol and rifampicin. Strains were categorized as susceptible or resistant based on European Committee on Antimicrobial Sensitivity Testing breakpoint cut‐offs assigned using published criteria.^17^

### Statistical analysis

Statistical analysis was undertaken using algorithms within Stata 14.2 (StataCorp, College Station, TX, U.S.A.). Comparisons of unpaired proportions were derived from a modified χ^2^‐test using the method described by Newcombe and Altman.^18^ To aid interpretation of the relevance, 95% confidence intervals (CIs) for observed differences in cases compared with controls are presented. The significance threshold for all analyses was set at 0·05. Each of the comparisons was decided beforehand; we did not statistically adjust for multiple comparisons. All testing was two‐tailed.

## Results

### Genetic backgrounds of *Staphylococcus aureus* from cases and controls

Ninety‐nine *S. aureus* isolates, 50 from cases of AD and 49 from nasal carriage controls, underwent AMS testing and whole genome sequencing. The participant demographics are summarized in Table S1 (see Supporting Information). Genomic analysis revealed a diverse collection, with 19 individual sequence types (STs) from 10 clonal complexes (CCs) in cases, and 16 STs representing nine CCs in controls. Comparison of case and control isolates demonstrated that they were comprised of several dominant clones (Table 1). In cases, CC1 isolates were the single most prevalent, accounting for 20% of samples, compared with 8% of controls. Isolates belonging to CC30 and CC45 predominated in controls, making up 33% and 22% of samples, respectively, compared with 10% and 14%, respectively, in cases. Isolates from CC7, CC9 and CC59 were identified only in cases, whereas CC22 and CC25 isolates were present only within controls.

**Table 1:** Comparison of the clonal backgrounds of strains colonizing either cases of atopic dermatitis (AD) or nasal carriage (NC) controls. Singleton isolates that do not fall within a defined clonal complex are presented as per their multilocus sequence type

Clonal complex (CC) or sequence type (ST) of colonizing strain	Cases of AD, *n* (%)	NC controls, *n* (%)
CC1	10 (20)	4 (8)
CC5	6 (12)	8 (16)
CC7	3 (6)	0
CC8	7 (14)	1 (2)
CC9	3 (6)	0
CC15	3 (6)	1 (2)
CC22	0	5 (10)
CC25	0	1 (2)
CC30	5 (10)	16 (33)
CC45	7 (14)	11 (22)
CC59	3 (6)	0
CC121	1 (2)	1 (2)
ST779	1 (2)	1 (2)
ST1290	1 (2)	0

### Distribution of antibiotic resistance phenotypes

From AMS testing, the average number of resistances per isolate between cases and controls did not differ significantly between the groups, with 1·5 antibiotics per isolate in AD and 1·3 in controls. Penicillin resistance was the most common among all isolates; 92% were resistant to this beta‐lactam antibiotic. Comparison demonstrated that penicillin resistance was less frequent in cases than in controls, present in 86% of AD and 98% of control isolates (95% CI for difference −22% to 2%, *P* = 0·029). Prevalence of MRSA was low generally, in 4% and 2% of cases and controls, respectively (95% CI for difference −5% to 9%, *P* = 0·57).

Between cases and controls there was no detectable difference in resistance to either the macrolide antibiotic erythromycin or the lincosamide clindamycin, exhibited by 12% of AD isolates compared with 6% of controls (95% CI for difference −5% to 17%, *P* = 0·31). Tetracycline resistance was less frequent in cases than in controls, but this was not statistically significant (4% vs. 10%; 95% CI for difference −4% to 16%, *P* = 0·23). A single case sample was resistant to both ciprofloxacin and gentamicin, while a single control was trimethoprim resistant. None of the isolates was resistant to vancomycin, daptomycin, linezolid, chloramphenicol, rifampicin or teicoplanin (Table S1; see Supporting Information).

Resistance to fusidic acid, which is widely used topically for superficial skin infections and in AD with topical corticosteroids, clearly differentiated the populations, with 24% more AD isolates than controls exhibiting resistance (95% CI for difference 6–41%, *P* = 0·009). Resistance to mupirocin, used topically and commonly for MRSA decolonization, was present in single isolates from each group (95% CI for difference −6% to 6%, *P* = 0·99).

### Genetic basis of antimicrobial resistance

Whole genome sequencing of the isolates allowed us to obtain a high‐resolution view of the population structure of *S. aureus* from cases and controls, to pinpoint the genetic basis of resistance and reconstruct their evolutionary context.

*In silico* characterization of the isolates’ resistome revealed resistance determinants for penicillin (*blaZ*), methicillin (*mecA*), erythromycin (*ermA*,* ermC*), tetracycline (*tetK*,* tetM*), ciprofloxacin (mutation of *gyrA*, S84L, and *grlA*, S80F), gentamicin (*aacA‐phD*), trimethoprim (*dfrG*) and mupirocin (mutation of *ileS‐1*, V588F) (Table S1; see Supporting Information). The resistance phenotype and genotype were concordant, with four exceptions, all of which were associated with penicillin resistance, where *blaZ* was detected but the isolates were sensitive to this beta‐lactam antibiotic. Closer examination of the sequence revealed that two isolates contained frameshift mutations within *blaZ* and two contained frameshifts in the regulatory gene *blaR* (which is responsible for expression of *blaZ*), both of which would ablate expression of *blaZ*.

Additionally we identified genes for resistance to antibiotics not commonly used for treatment in AD, or routinely incorporated in AMS testing. Streptomycin resistance markers (*AAD9* or *aadE*) were found in 12% of cases of AD vs. 6% of controls. The amikacin resistance gene *aphA‐3* was detected in 4% of *S. aureus* from cases compared with 2% from controls. However, overall there were no significant differences in these genes between the groups.

Finally, we assessed the WGS data for determinants of resistance to disinfectants. In 16% of the *S. aureus* isolates from cases of AD we identified *qac* genes, compared with 2% from controls (95% CI for difference 3–25%, *P* = 0·016). These have been associated with reduced susceptibility to antiseptics such as chlorhexidine and benzalkonium chloride,^19^ which are commonly used in dermatological practice.

### Distribution of resistance genes

**Figure 1:**
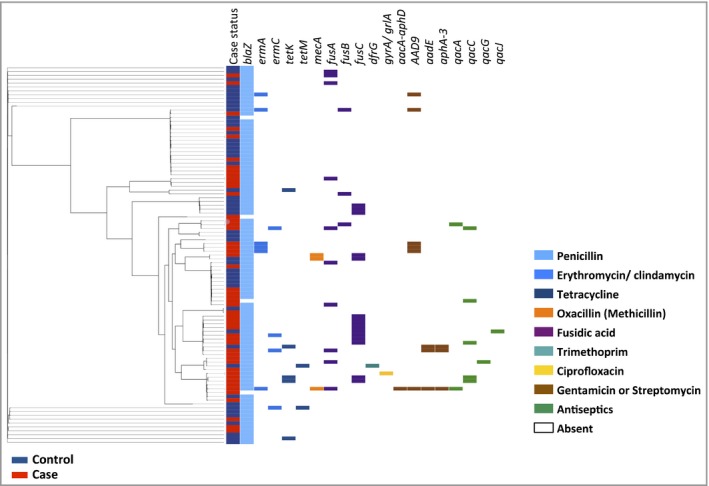
Resistome profile of atopic dermatitis (AD) case and control isolates. Maximum likelihood tree of 99 isolates (50 cases of AD, 49 nasal carrier controls) built with core genome single nucleotide polymorphisms. The case status of each isolate is indicated by the coloured cell (red, AD; blue, nasal carrier control). Coloured cells then indicate the presence of resistance determinants to the antimicrobial agents penicillin (*blaZ*), erythromycin (*ermA*,* ermC*), tetracyclines (*tetK*,* tetM*), methicillin (*mecA*), fusidic acid (*fusB* and *fusC*, mutations in *fusA*), trimethoprim (*dfrG*), ciprofloxacin (mutations in *gyrA*/*grlA*), aminoglycosides (gentamicin, *aacA‐aphD*; streptomycin, *AAD9*,* aadE*,* aphA‐3*) and antiseptics (*qacA*,* qacC*,* qacG* and *qacJ*). Blank cells indicate that the gene or mutation is absent.

We examined the distribution of antibiotic resistance determinants within the population framework generated from the core‐genome phylogenetic analysis (Fig. 1). The penicillin resistance gene *blaZ* was present in 94% of the *S. aureus* from cases and 98% from controls, reflecting the widespread distribution of beta‐lactamases in the *S. aureus* population generally. Of three *mecA*‐carrying isolates (two cases and one control), two belonged to ST779 (one case and one control) and one belonged to ST8. The *ermA* and *ermC* genes, which confer resistance to both erythromycin and clindamycin, were found in both patient groups, with *ermA* being more frequent in AD samples, as expected given its high level of carriage in CC9 isolates, a CC present only in cases.

Tetracycline resistance genes *tetK* and *tetM* were both present in control isolates of multiple clonal backgrounds, while *tetK* was sporadically present in CC8 case isolates. Both mupirocin‐resistant isolates had the same point mutation in *ileS‐1*, but from differing clonal backgrounds, demonstrating that they arose independently. Finally, the *qac* genes are seen scattered throughout the population in multiple genetic backgrounds. Taken as a whole, the distribution of these determinants varied across the population, and cases and controls could not be segregated on the basis of their resistome.

### Genetic basis of fusidic acid resistance

Phenotypic analysis suggested that fusidic acid resistance (Fus^R^) was significantly associated with AD. Three genotypes responsible for Fus^R^ were identified, including acquired genes *fusB* and *fusC* and chromosomal mutations in the gene *fusA*.

**Figure 2:**
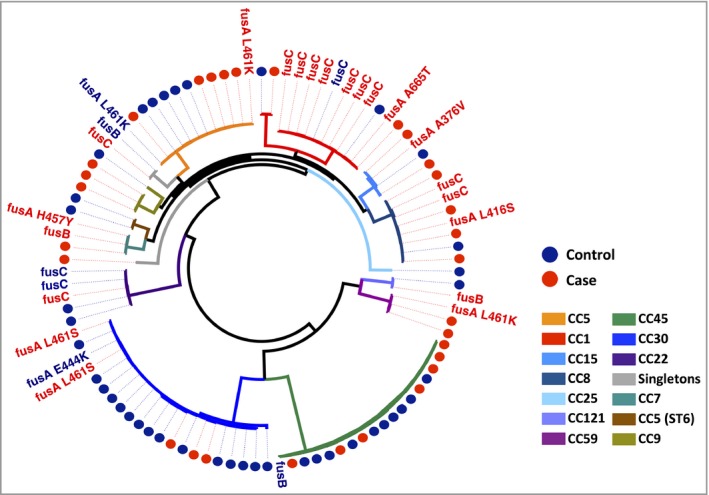
Distribution of fusidic acid resistance determinants within the population. Maximum likelihood core phylogeny of all cases of atopic dermatitis (AD) and nasal carrier (NC) controls. The branch colouring corresponds to the clonal background of the isolates. Taxon labels are coloured according to case status (AD case red, NC control blue). Fusidic acid sensitive isolates are labelled as coloured circles (AD case red, NC control blue). Resistant isolates are labelled according to the genetic determinant. In isolates with multiple *fusA* mutations, a single mutation is presented on the branch, corresponding to either a previously reported amino acid change conferring resistance, or the most common. Singleton isolates fall out with a defined clonal complex and include ST779 and ST1290.

Overall *fusB* was the least prevalent Fus^R^ determinant, found in 4% of cases compared with 2% of controls (95% CI for difference −5% to 9%, *P* = 0·57). Carriage of *fusC* was detected in 20% of cases compared with 10% of controls (95% CI for difference −4% to 24%, *P* = 0·17), and predominantly in CC1 isolates (Fig. 2). Similarly, the difference in the proportion of fusC‐positive CC1 isolates between cases and controls was not significant (95% CI for difference −6% to 96%, *P* = 0·12). Point mutations in *fusA* were fourfold higher in cases than in controls (16% vs. 4%; 95% CI for difference 0–23%, *P* = 0·049). In total 12 mutations responsible for resistance were identified in 10 resistant isolates, with four AD isolates having multiple mutations (Table 1). Mutations in codon 461 of *fusA*, responsible for an amino acid substitution leucine to serine at this position, were the most frequent (*n* = 4).

The phenotypic resistance observed varied depending on the genetic determinant the isolate possessed, with the highest level of resistance associated with *fusA* mutations (Table 2). High‐level resistance (minimum inhibitory concentration > 32 μg mL^−1^) in *fusA* mutants was detected in five isolates (four cases, one control). As shown in Figure 2, the same *fusA* mutations were distributed in multiple CCs, suggesting that they evolved independently, for example the substitution L461S in CC8 and CC30 samples. This indicates that the prevalence of Fus^R^ was not driven by expansion of one successful clone, but rather by development of resistance in multiple clones, on multiple occasions, suggesting that a strong selective pressure has been exerted by fusidic acid on the population.

**Table 2:** Fusidic acid resistance (Fus^R^) determinants identified in case and control isolates, and associated minimum inhibitory concentrations (MICs)

Fus^R^ determinant	Amino acid substitution	Number of isolates (case status)	Fusidic acid MIC (mg mL^−1^)
*fusA*	A307S,^a^ L461S	1 (AD)	4
*fusA*	L461S	2 (AD)	4
*fusA*	E444K	1 (NC)	4
*fusA*	V90I, A655T^b^	1 (AD)	16
*fusA*	A376V, P40Q, L461S	1 (AD)	> 32
*fusA*	L461K	3 (2 AD, 1 NC)	> 32
*fusA*	T34S,^a^ D283N,^a^ H457Y, P635L^a^	1 (AD)	> 32
*fusB*	n/a	3 (2 AD, 1 NC)	8–16
*fusC*	n/a	15 (10 AD, 5 NC)	8–16

AD, case of atopic dermatitis; NC, nasal carriage control; n/a, not applicable. ^a^Nonsynonymous mutation without previously published reports of impact of mutation on resistance. ^b^Mutation at this codon previously reported but with different amino acid substitution.^36^

## Discussion

The association between disease activity and *S. aureus* means that antimicrobials are frequently used in patients with AD. Increasingly it is becoming evident that there are specific lineages seemingly adapted to colonizing and surviving on AD‐affected skin.^20^ This analysis has demonstrated differences in genetic backgrounds of *S. aureus* colonizing patients with AD compared with controls, and a marked difference in the prevalence of topical antimicrobial resistance determinants among children with AD.

Antimicrobial resistance is a concern in AD, often with specific emphasis being placed on MRSA.^21^, ^22^, ^23^ Our results demonstrated that MRSA prevalence in cases and controls was low, just 4% and 2%, respectively. This reflects the population prevalence of MRSA in this geographical locality where previous screening found MRSA in 1·6% of children aged < 18 years (Désireé Bennett, personal communication). Intriguingly, penicillin resistance was more common in control isolates (98%, vs. 86% in cases). While this difference appears statistically significant, we hypothesize that assessment of a larger sample size would void this difference, as penicillin sensitivity in *S. aureus* in Europe and North America reportedly varies between 8% and 13%.^24^, ^25^ Erythromycin resistance was twice as common in cases than in controls, but numbers were small and this difference might have been a chance finding. It is worth noting that macrolides are the usual alternative to first‐line penicillin‐based agents for penicillin‐allergic individuals with AD flare; it is possible that with a much larger study this difference may have been significant.

The relevance of the significantly greater prevalence of the *qac* genes in cases of AD is uncertain. However, given the widespread use of antiseptics in dermatology, it may be functionally important. The reasoning for our cautious interpretation of this finding is the lack of clear genotype–phenotype correlation with regards to the carriage of *qac* genes, as well as issues surrounding the lack of standardized testing methods for antiseptic susceptibility.^26^ Nonetheless, the potential for them to function in reducing susceptibility to antiseptic compounds used in AD warrants investigation.

From our analysis of antibiotic resistances between cases and controls, the strongest signal of antibiotic selection came from fusidic acid. This is among the most common interventions in AD, principally in the community in the U.K. and Ireland. Resistance was 2·5 times more frequent in cases, and displayed greater diversity in the genetic determinants responsible for it. Rates of Fus^R^ in *S. aureus* vary depending upon country and the patient population sampled. One European surveillance survey showed Fus^R^ in 11·8% of isolates from the U.K., while in Ireland this rate was higher at 19·9%.^27^ This is in contrast with the U.S.A., where fusidic acid is not routinely used, and sensitivity rates of 99·6% are reported.^28^ Higher rates of resistance have been shown specifically within dermatology patients, believed to be directly influenced by usage of topical fusidic acid preparations.^29^ Conversely, resistance to mupirocin, another topical anti‐staphylococcal, was low in both groups, likely because of comparatively low usage in Ireland.

Mechanistically, fusidic acid inhibits bacterial protein synthesis through binding to translation elongation factor G (*fusA*), a GTPase catalysing the final stage of peptide elongation. Resistance arises either via acquisition of a plasmid‐derived determinant or through point mutations in *fusA*. Two acquired genes (*fusB* or *fusC*) and six nonsynonymous substitutions were identified in the isolates. Placing these in phylogenetic context, we estimate that Fus^R^ arose at least 18 times in the observed population. The basis of Fus^R^ also significantly differentiated the populations. Both plasmid‐derived Fus^R^ determinants were present twice as frequently in case isolates. Notably, *fusC* was found in 20% of cases, of which 70% were from CC1 isolates. This determinant has been reported in the context of its distribution in successful Fus^R^ clones belonging to CC1, both methicillin sensitive and resistant alike.^30^, ^31^

While *fusC* prevalence seems clonally influenced, the *fusA* mutations are indicative of prior exposure and adaptation to fusidic acid therapy. Numerous *fusA* SNPs were identified across the whole population (Fig. 2), demonstrating that this was the consequence of repeated independent events. Several case isolates had multiple mutations in *fusA*. Previously it has been reported that secondary mutations in *fusA* provide a potential mechanism to offset the fitness deficit incurred by maintaining this amino acid change.^32^

These observations raise several important points for clinical consideration. Firstly, do our prescribing practices at a population level select for specific colonizing strains in AD? Strain prevalence in AD is an emerging area of interest, and little is presently understood about the genetic basis of the preferential success of certain lineages, but this study supports the recent findings of strain preponderance.^20^ Secondly, does patient behaviour in addition to prescribing practice contribute to the accumulation of *fusA* mutations in cases? Anecdotally, patients often report using repeated short bursts of fusidic acid preparations at home for disease flares. Several studies have that suggested both intermittent and prolonged usage of such therapies is very likely to contribute to the development of resistance.^30^, ^33^ The patients with AD in this study were attending a tertiary clinic for the first time, and will likely have received this antibiotic in the community.

One limitation of this study was the lack of detailed prescribing records for the participants. The results nonetheless highlight the importance of antimicrobial stewardship in this specific disease context. Finally we have to consider whether the use of any antibiotic is warranted in many cases of AD flare. Recent clinical trial evidence has clearly demonstrated a lack of objective benefit of antimicrobials over use of a moderate‐potency topical steroid, at least in mild disease exacerbation.^34^, ^35^

Future studies are specifically needed to assess the impact of antimicrobial usage on *S. aureus* populations in AD. Topical antimicrobials, both antibiotics and antiseptics, are of particular interest. These studies must incorporate both community‐based patients and those under specialist dermatological care, and correlate with prescribing data. Patients of different ages must be assessed to allow examination of the selective impact of prescribing in dermatological and wider clinical practice. With increasing evidence of lack of benefit of these treatments, and growing resistance, we must reassess and change our clinical practice accordingly.

## Supporting information


**Table S1** Participant and isolate information.

**Powerpoint S1** Journal Club Slide Set.

**Video S1** Author Video.
