# Supplementary material for: The widespread use of topical antimicrobials enriches for resistance in Staphylococcus aureus isolated from patients with atopic dermatitis
Source: Br J Dermatol. 2018 Jul 24;179(4):951–8. doi: 10.1111/bjd.16722 (PMC6221151; doi:10.1111/bjd.16722)
Supplement: Supplementary file 2 — Powerpoint S1 Journal Club Slide Set. [file BJD-179-951-s002.pptx]

## Slide 1
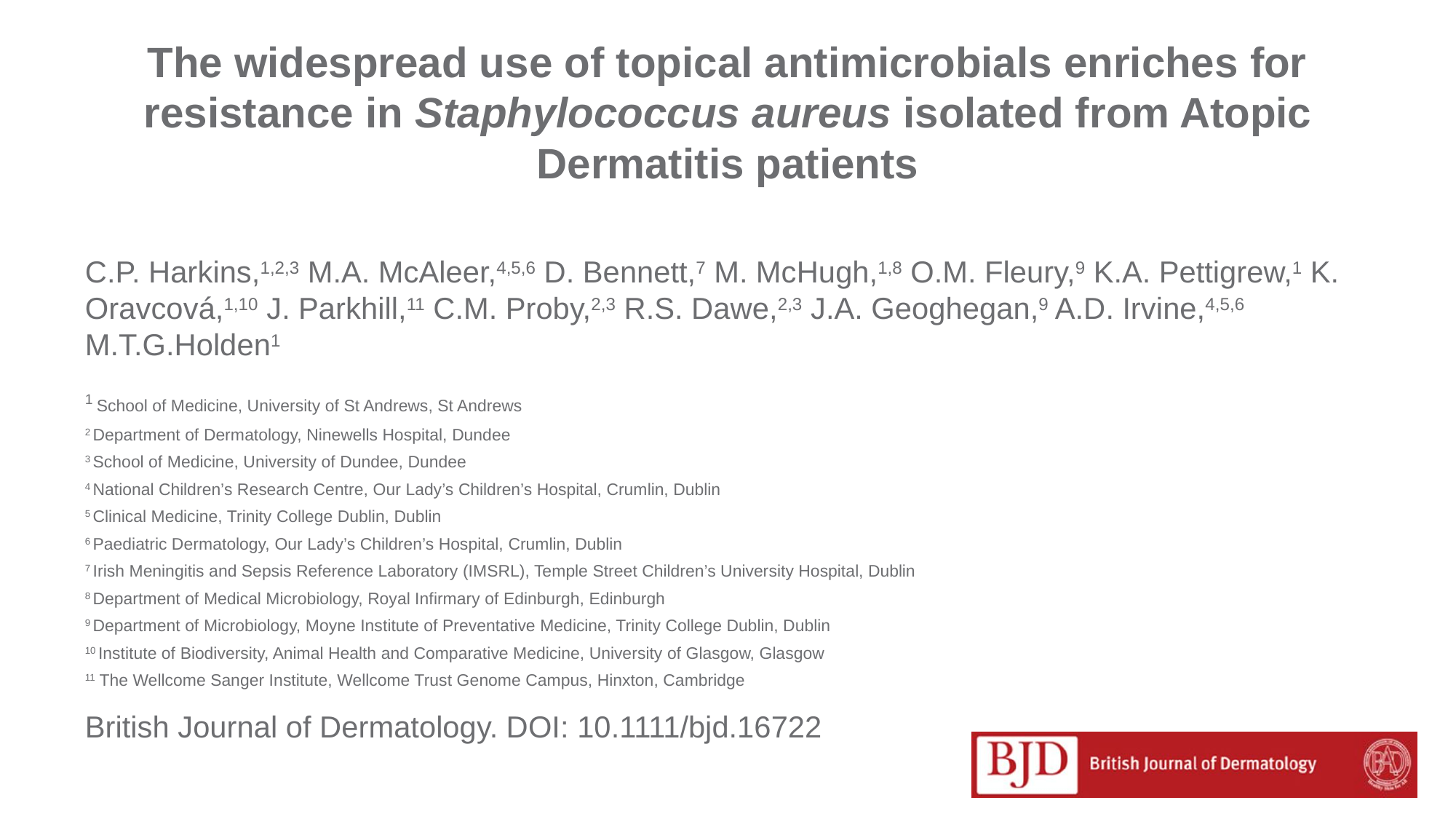

# The widespread use of topical antimicrobials enriches for resistance in Staphylococcus aureus isolated from Atopic Dermatitis patients
C.P. Harkins,1,2,3 M.A. McAleer,4,5,6 D. Bennett,7 M. McHugh,1,8 O.M. Fleury,9 K.A. Pettigrew,1 K. Oravcová,1,10 J. Parkhill,11 C.M. Proby,2,3 R.S. Dawe,2,3 J.A. Geoghegan,9 A.D. Irvine,4,5,6 M.T.G.Holden1
1 School of Medicine, University of St Andrews, St Andrews
2 Department of Dermatology, Ninewells Hospital, Dundee
3 School of Medicine, University of Dundee, Dundee
4 National Children’s Research Centre, Our Lady’s Children’s Hospital, Crumlin, Dublin
5 Clinical Medicine, Trinity College Dublin, Dublin
6 Paediatric Dermatology, Our Lady’s Children’s Hospital, Crumlin, Dublin
7 Irish Meningitis and Sepsis Reference Laboratory (IMSRL), Temple Street Children’s University Hospital, Dublin
8 Department of Medical Microbiology, Royal Infirmary of Edinburgh, Edinburgh
9 Department of Microbiology, Moyne Institute of Preventative Medicine, Trinity College Dublin, Dublin
10 Institute of Biodiversity, Animal Health and Comparative Medicine, University of Glasgow, Glasgow
11 The Wellcome Sanger Institute, Wellcome Trust Genome Campus, Hinxton, Cambridge
British Journal of Dermatology. DOI: 10.1111/bjd.16722

## Slide 2
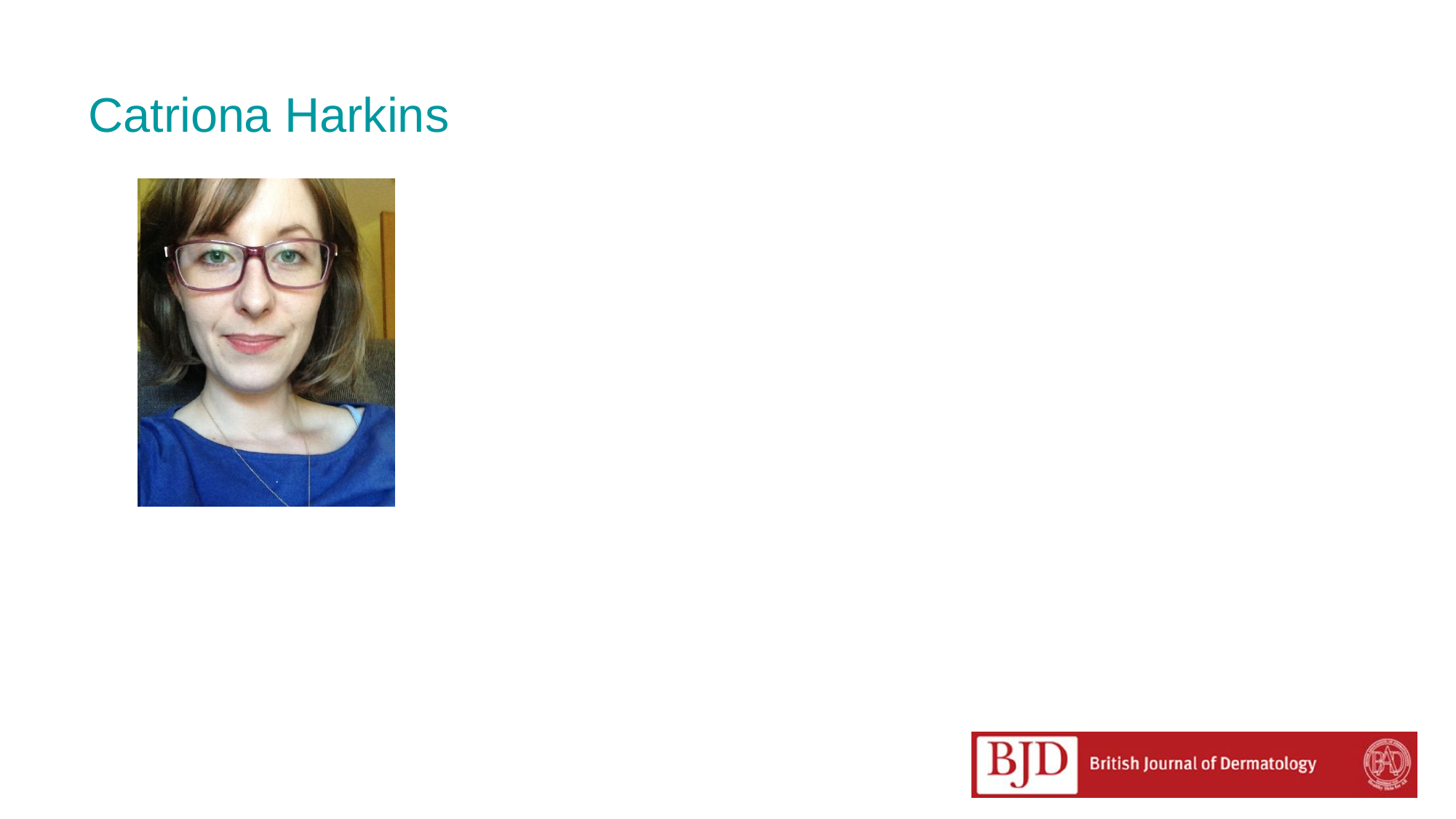

# Catriona Harkins

## Slide 3
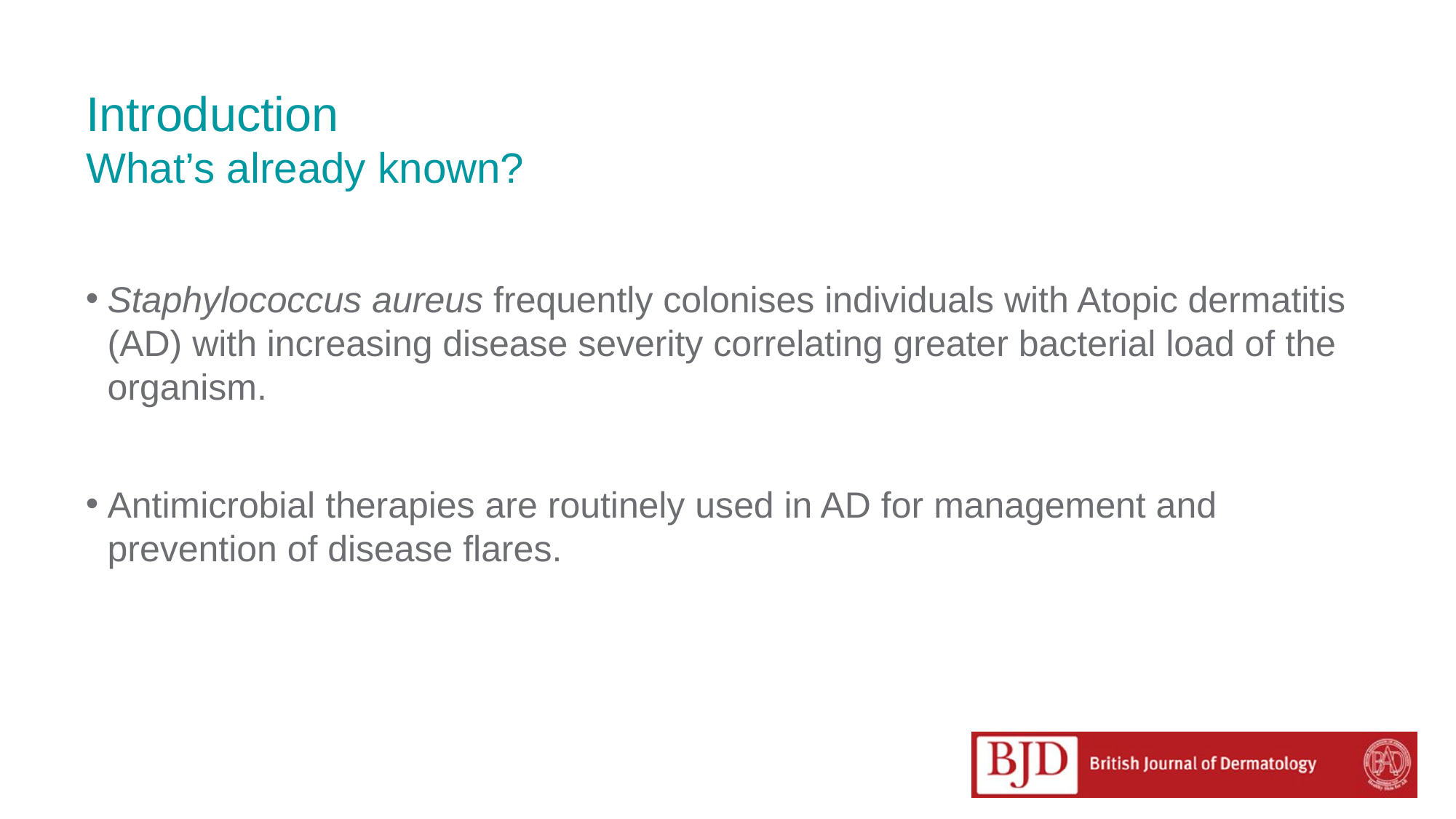

# Introduction What’s already known?
Staphylococcus aureus frequently colonises individuals with Atopic dermatitis (AD) with increasing disease severity correlating greater bacterial load of the organism.
Antimicrobial therapies are routinely used in AD for management and prevention of disease flares.

## Slide 4
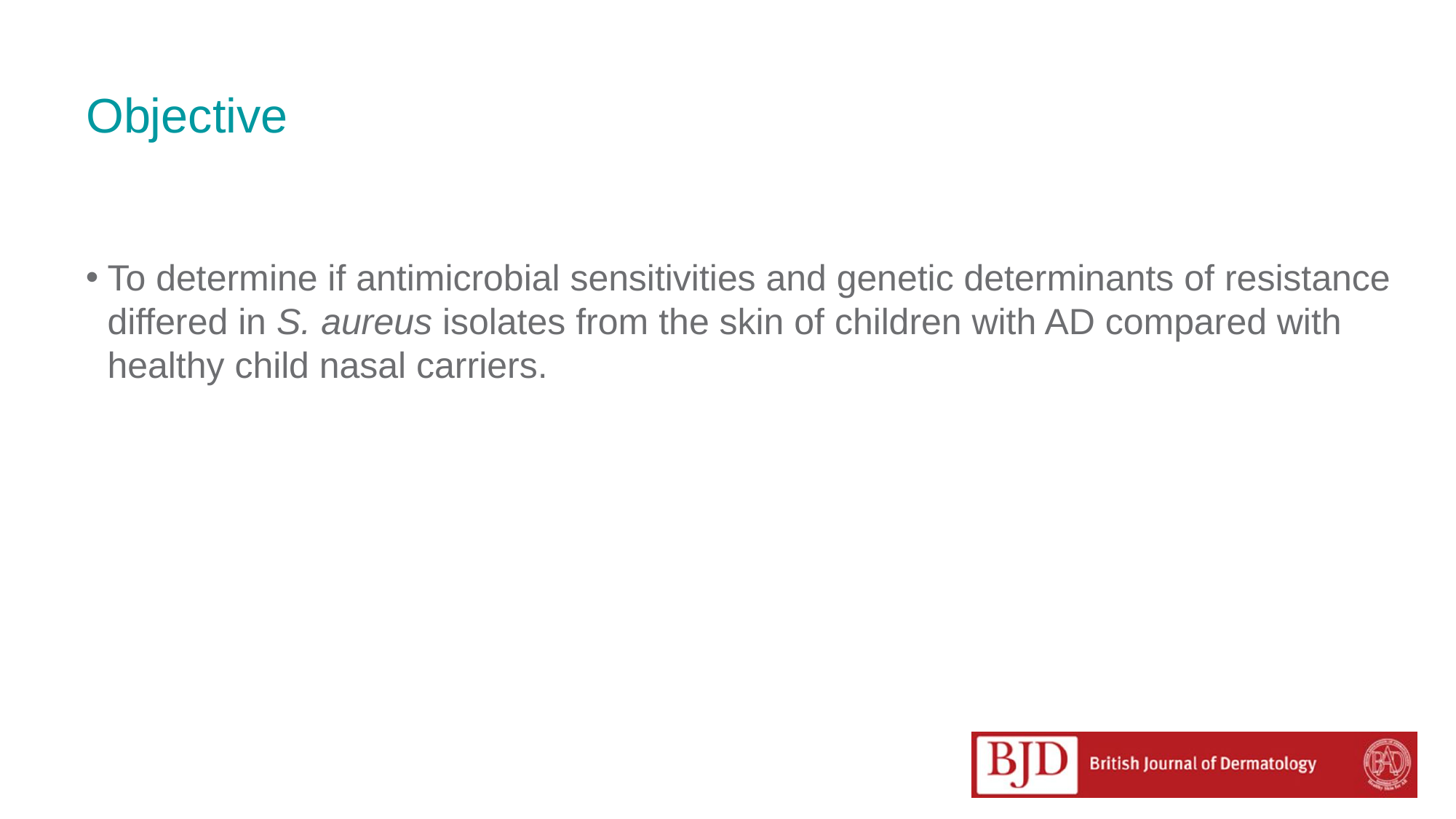

# Objective
To determine if antimicrobial sensitivities and genetic determinants of resistance differed in S. aureus isolates from the skin of children with AD compared with healthy child nasal carriers.

## Slide 5
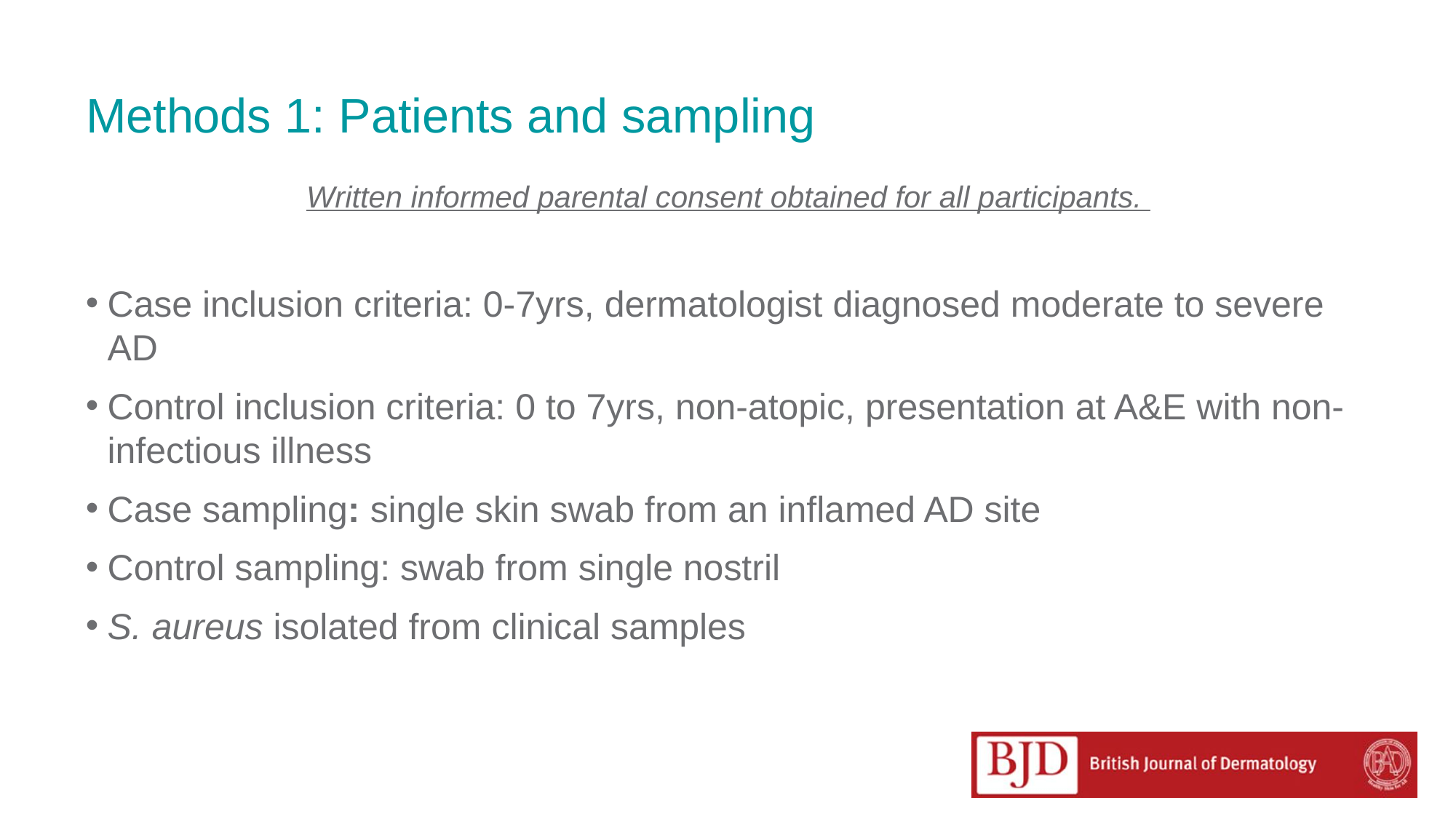

# Methods 1: Patients and sampling
Written informed parental consent obtained for all participants.
Case inclusion criteria: 0-7yrs, dermatologist diagnosed moderate to severe AD
Control inclusion criteria: 0 to 7yrs, non-atopic, presentation at A&E with non-infectious illness
Case sampling: single skin swab from an inflamed AD site
Control sampling: swab from single nostril
S. aureus isolated from clinical samples

## Slide 6
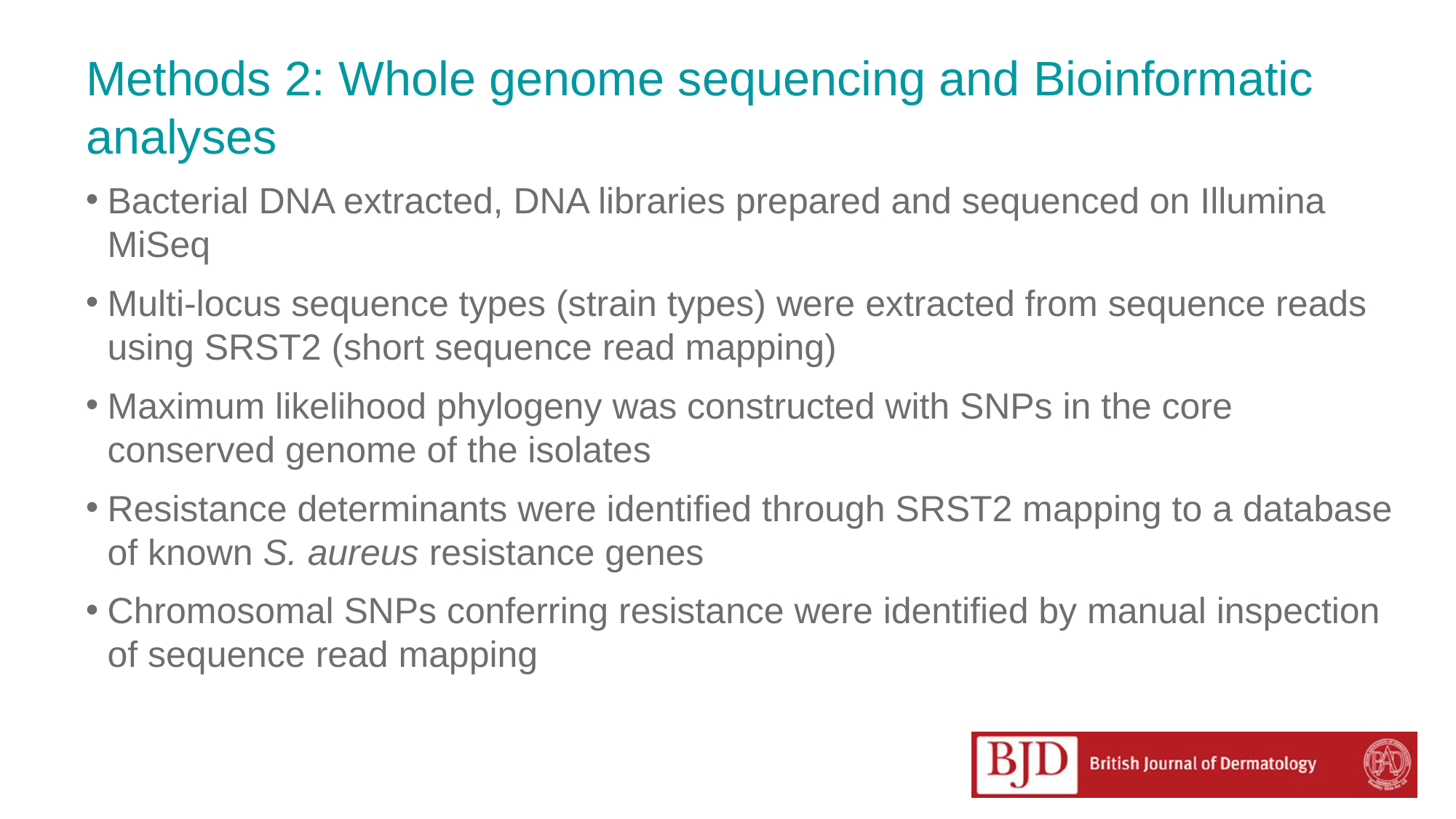

# Methods 2: Whole genome sequencing and Bioinformatic analyses
Bacterial DNA extracted, DNA libraries prepared and sequenced on Illumina MiSeq
Multi-locus sequence types (strain types) were extracted from sequence reads using SRST2 (short sequence read mapping)
Maximum likelihood phylogeny was constructed with SNPs in the core conserved genome of the isolates
Resistance determinants were identified through SRST2 mapping to a database of known S. aureus resistance genes
Chromosomal SNPs conferring resistance were identified by manual inspection of sequence read mapping

## Slide 7
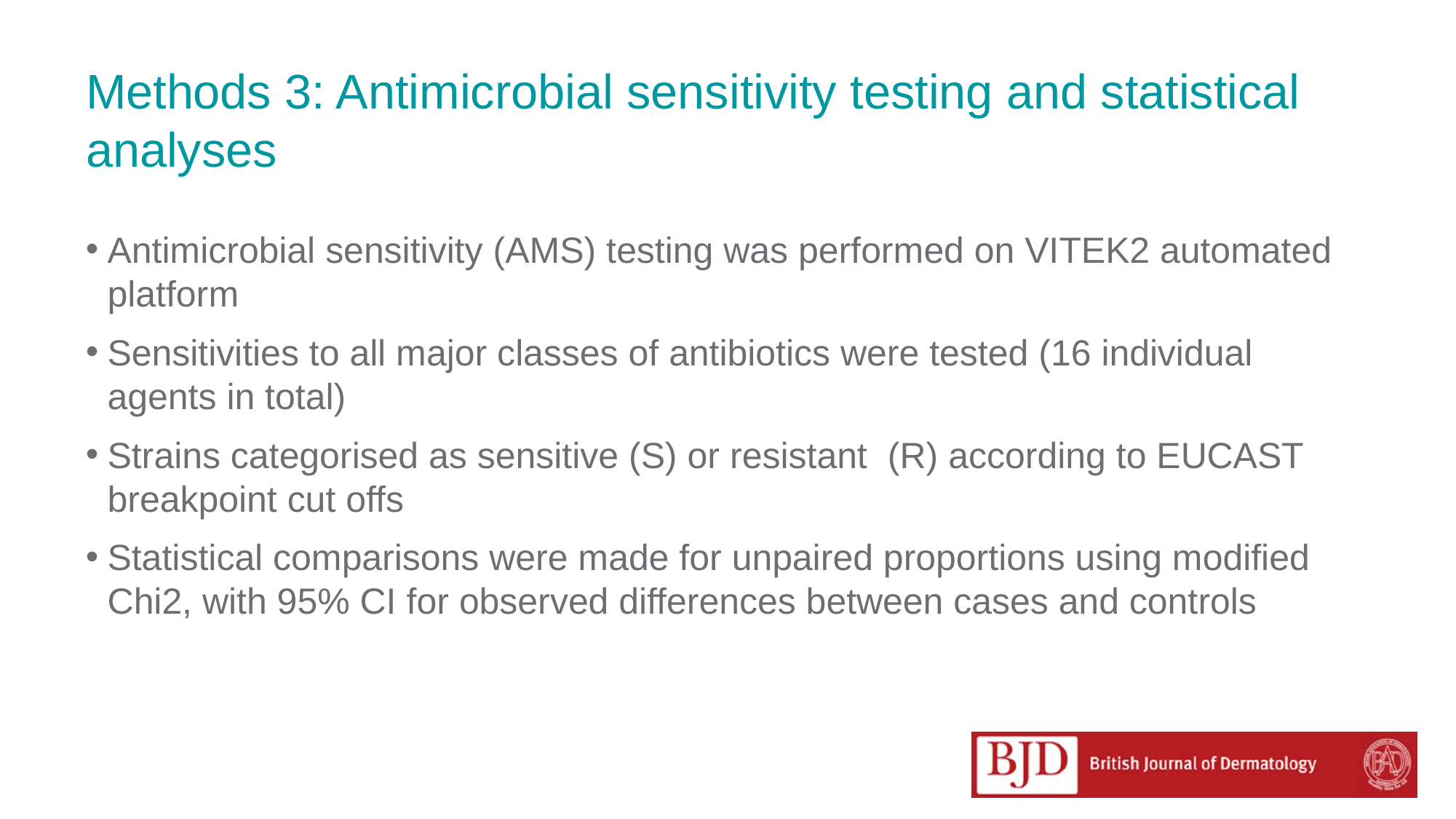

# Methods 3: Antimicrobial sensitivity testing and statistical analyses
Antimicrobial sensitivity (AMS) testing was performed on VITEK2 automated platform
Sensitivities to all major classes of antibiotics were tested (16 individual agents in total)
Strains categorised as sensitive (S) or resistant (R) according to EUCAST breakpoint cut offs
Statistical comparisons were made for unpaired proportions using modified Chi2, with 95% CI for observed differences between cases and controls

## Slide 8
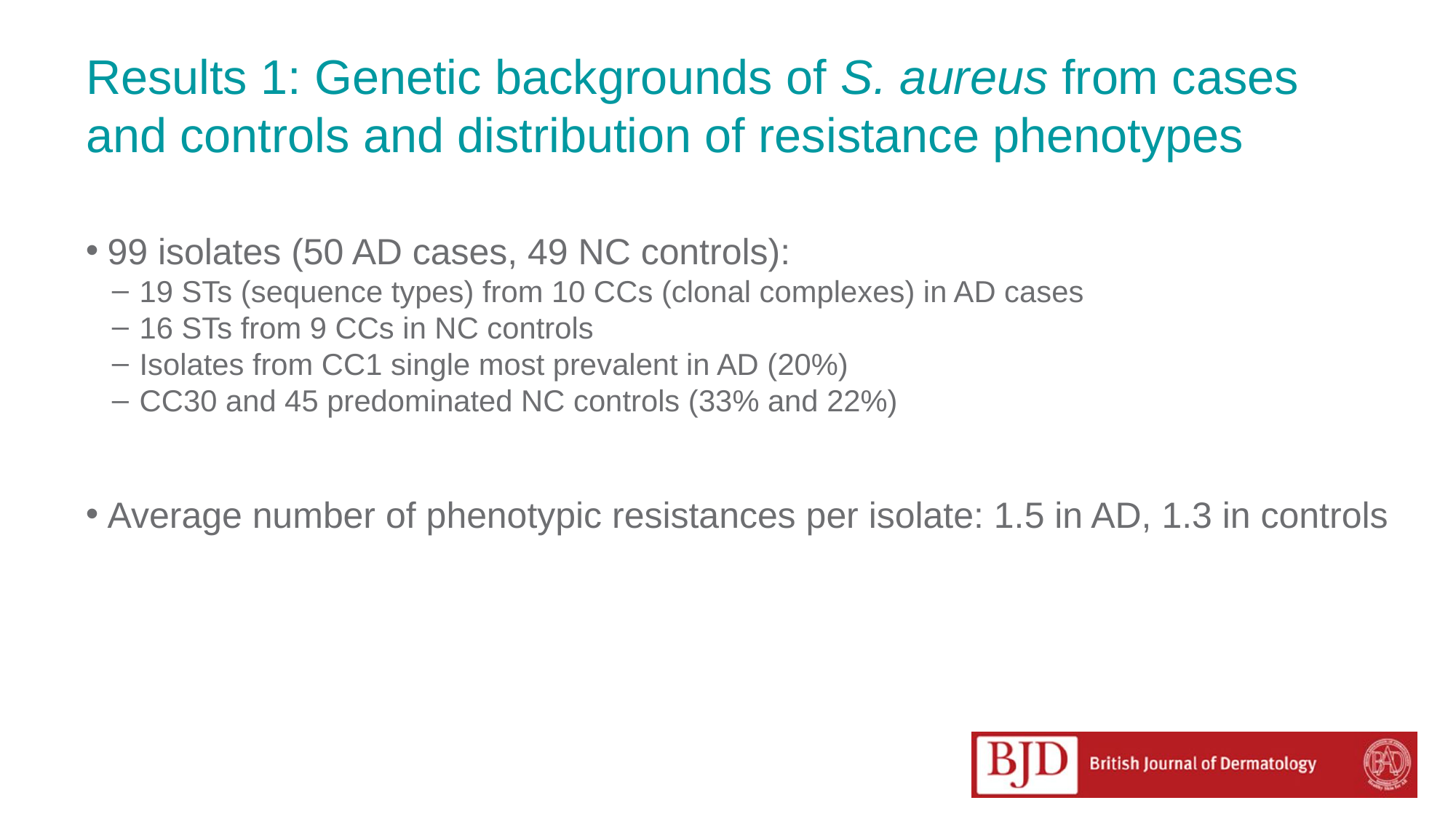

# Results 1: Genetic backgrounds of S. aureus from cases and controls and distribution of resistance phenotypes
99 isolates (50 AD cases, 49 NC controls):
19 STs (sequence types) from 10 CCs (clonal complexes) in AD cases
16 STs from 9 CCs in NC controls
Isolates from CC1 single most prevalent in AD (20%)
CC30 and 45 predominated NC controls (33% and 22%)
Average number of phenotypic resistances per isolate: 1.5 in AD, 1.3 in controls

## Slide 9
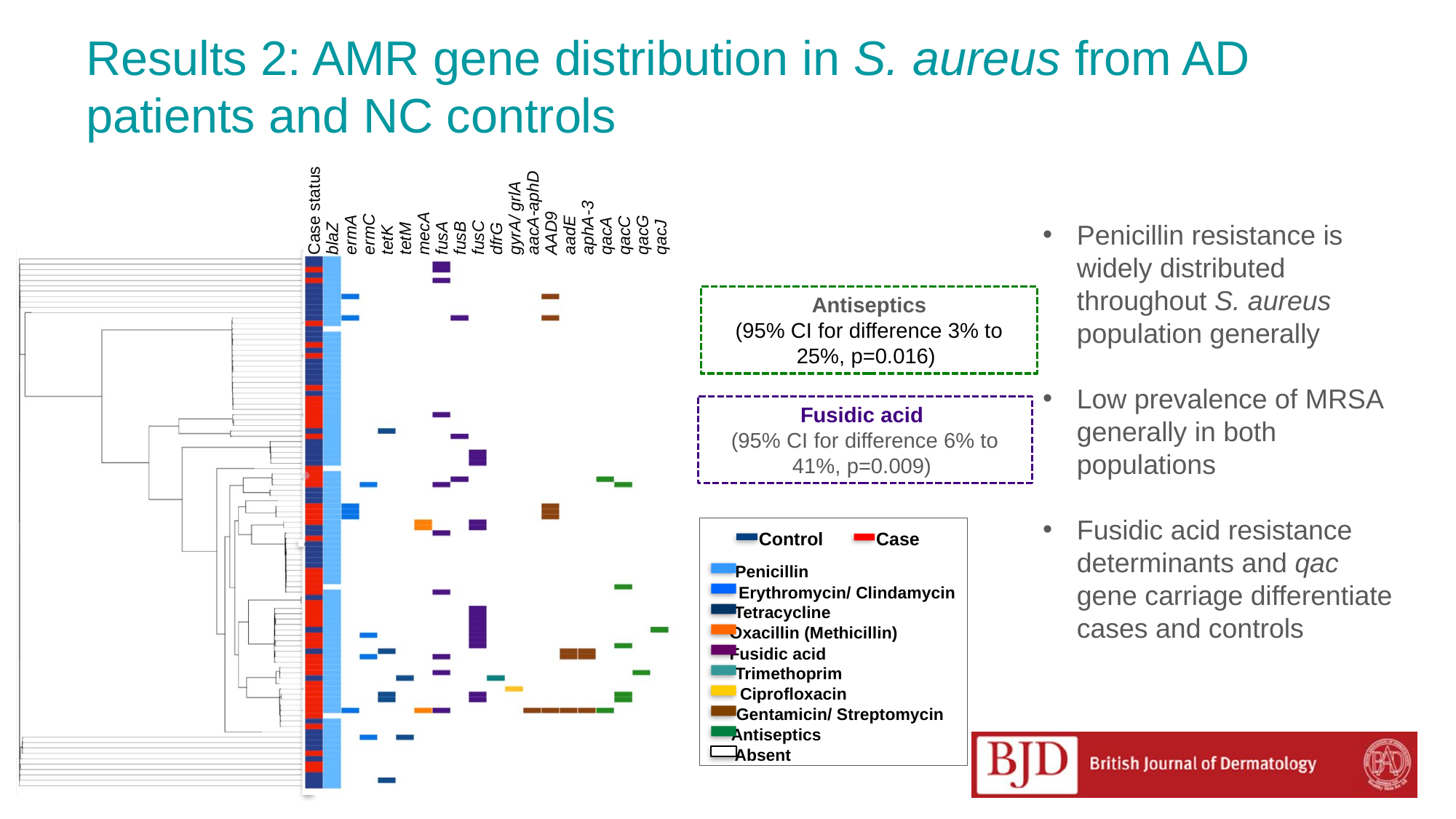

Case status
blaZ
ermA
ermC
tetK
tetM
mecA
fusA
fusB
fusC
dfrG
gyrA/ grlA
aacA-aphD
AAD9
aadE
aphA-3
qacA
qacC
qacG
qacJ
# Results 2: AMR gene distribution in S. aureus from AD patients and NC controls
Penicillin resistance is widely distributed throughout S. aureus population generally
Low prevalence of MRSA generally in both populations
Fusidic acid resistance determinants and qac gene carriage differentiate cases and controls
Antiseptics
(95% CI for difference 3% to 25%, p=0.016)
Fusidic acid
(95% CI for difference 6% to 41%, p=0.009)
Control
Case
Penicillin
Erythromycin/ Clindamycin
Tetracycline
Oxacillin (Methicillin)
Fusidic acid
Trimethoprim
Ciprofloxacin
Gentamicin/ Streptomycin
Antiseptics
Absent

## Slide 10
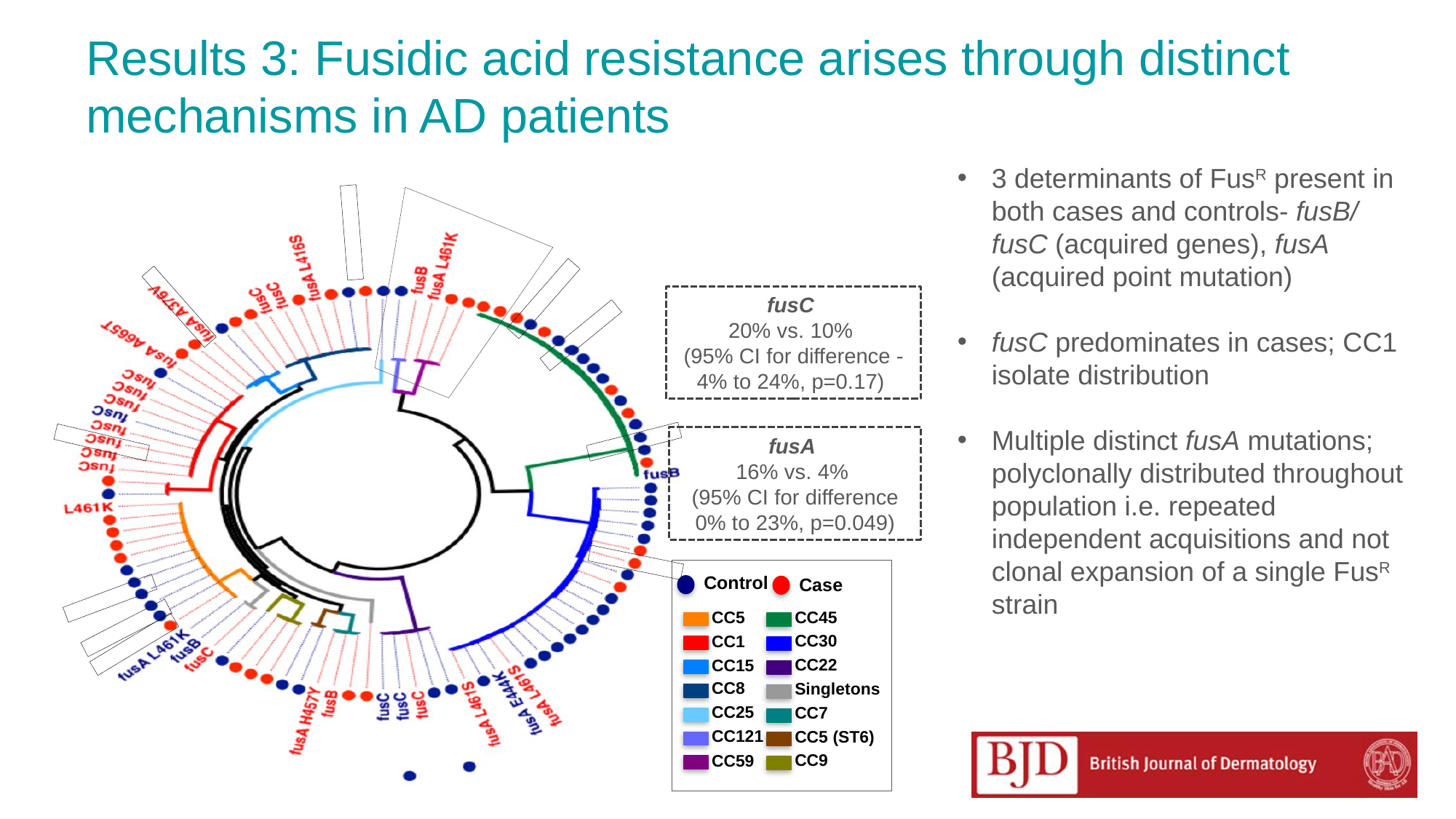

# Results 3: Fusidic acid resistance arises through distinct mechanisms in AD patients
3 determinants of FusR present in both cases and controls- fusB/ fusC (acquired genes), fusA (acquired point mutation)
fusC predominates in cases; CC1 isolate distribution
Multiple distinct fusA mutations; polyclonally distributed throughout population i.e. repeated independent acquisitions and not clonal expansion of a single FusR strain
fusC
20% vs. 10%
(95% CI for difference -4% to 24%, p=0.17)
fusA
16% vs. 4%
(95% CI for difference 0% to 23%, p=0.049)
Control
Case
CC5
CC1
CC15
CC8
CC25
CC121
CC59
CC45
CC30
CC22
Singletons
CC7
CC5 (ST6)
CC9

## Slide 11
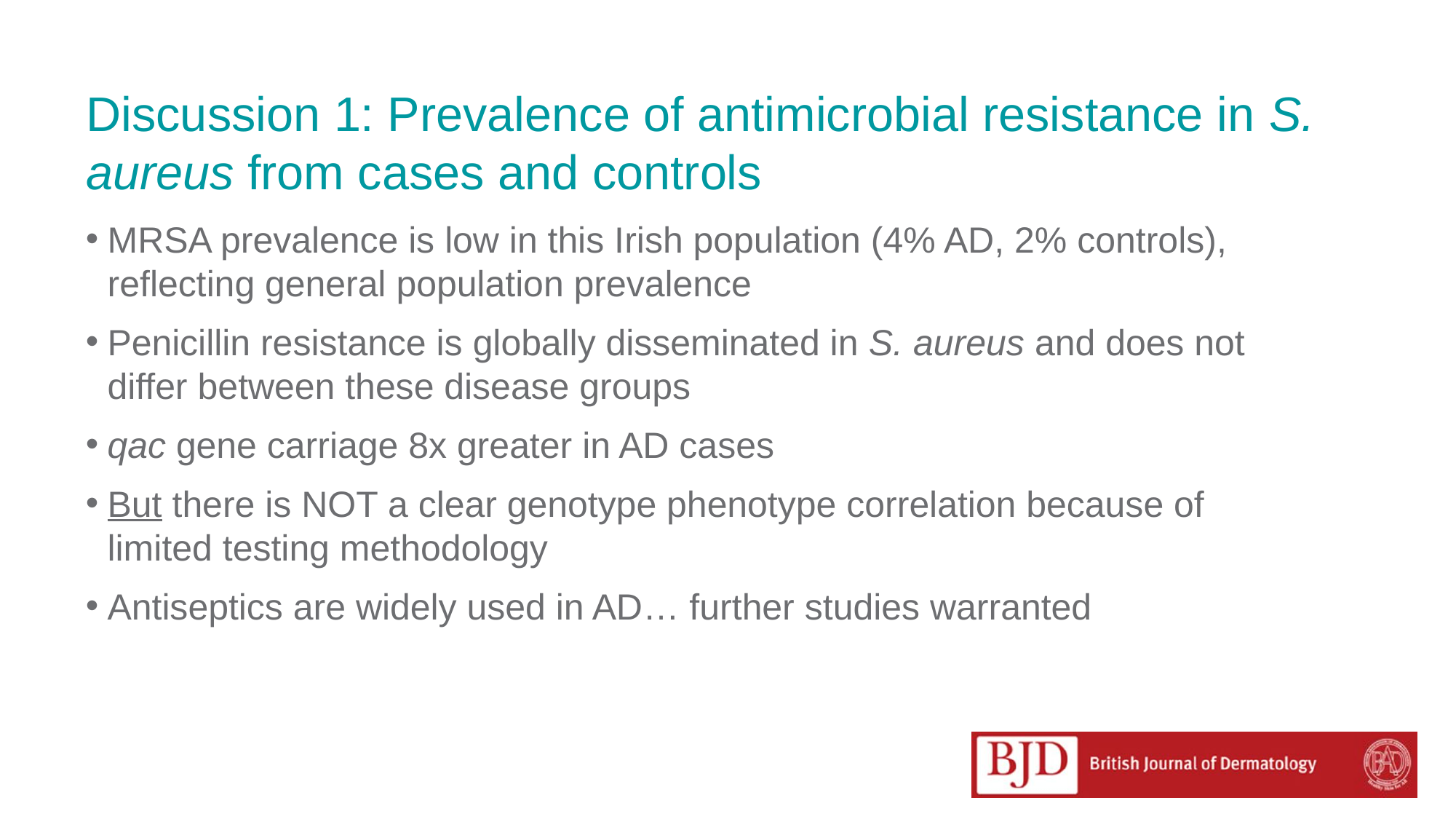

# Discussion 1: Prevalence of antimicrobial resistance in S. aureus from cases and controls
MRSA prevalence is low in this Irish population (4% AD, 2% controls), reflecting general population prevalence
Penicillin resistance is globally disseminated in S. aureus and does not differ between these disease groups
qac gene carriage 8x greater in AD cases
But there is NOT a clear genotype phenotype correlation because of limited testing methodology
Antiseptics are widely used in AD… further studies warranted

## Slide 12
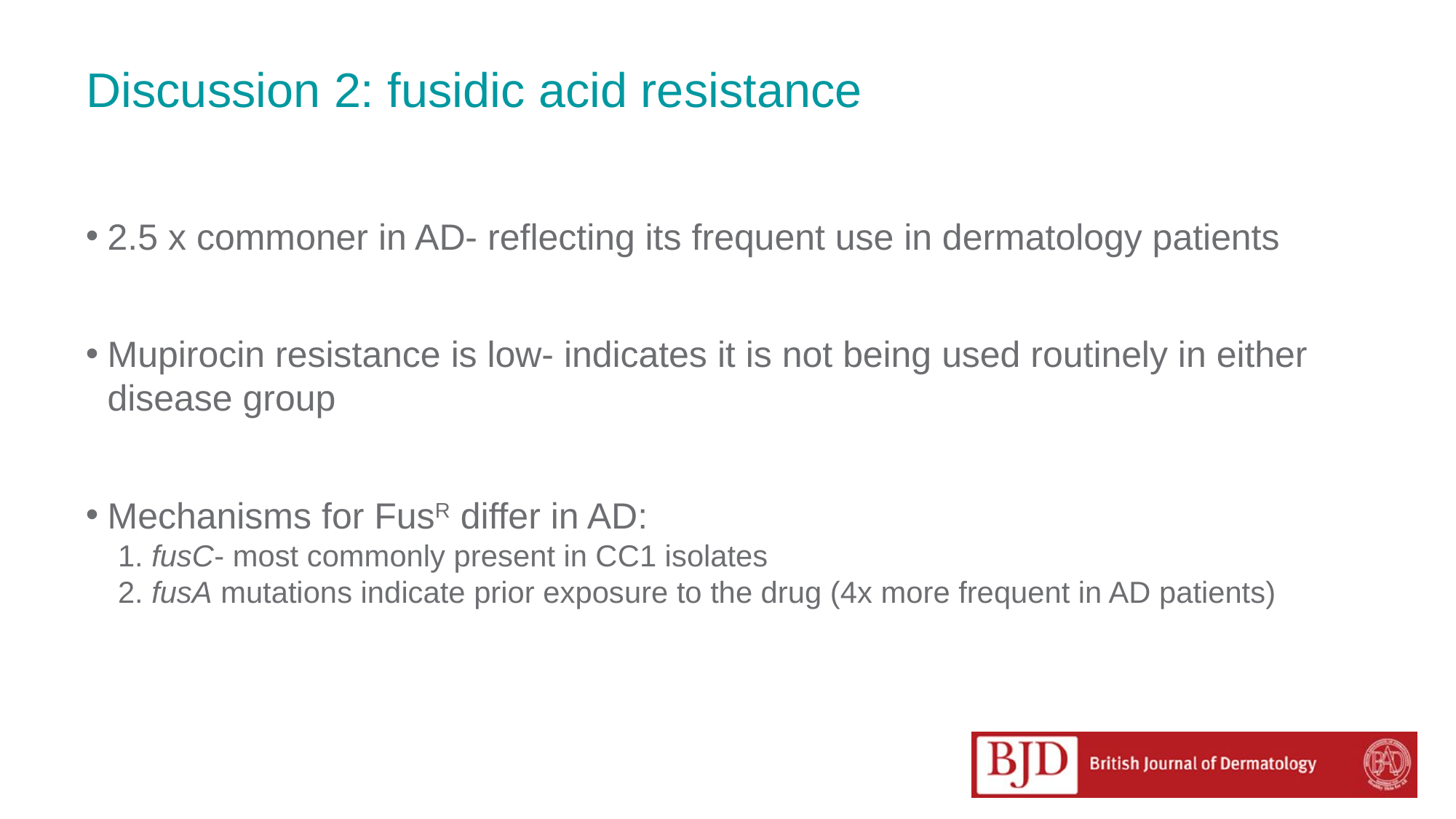

# Discussion 2: fusidic acid resistance
2.5 x commoner in AD- reflecting its frequent use in dermatology patients
Mupirocin resistance is low- indicates it is not being used routinely in either disease group
Mechanisms for FusR differ in AD:
1. fusC- most commonly present in CC1 isolates
2. fusA mutations indicate prior exposure to the drug (4x more frequent in AD patients)

## Slide 13
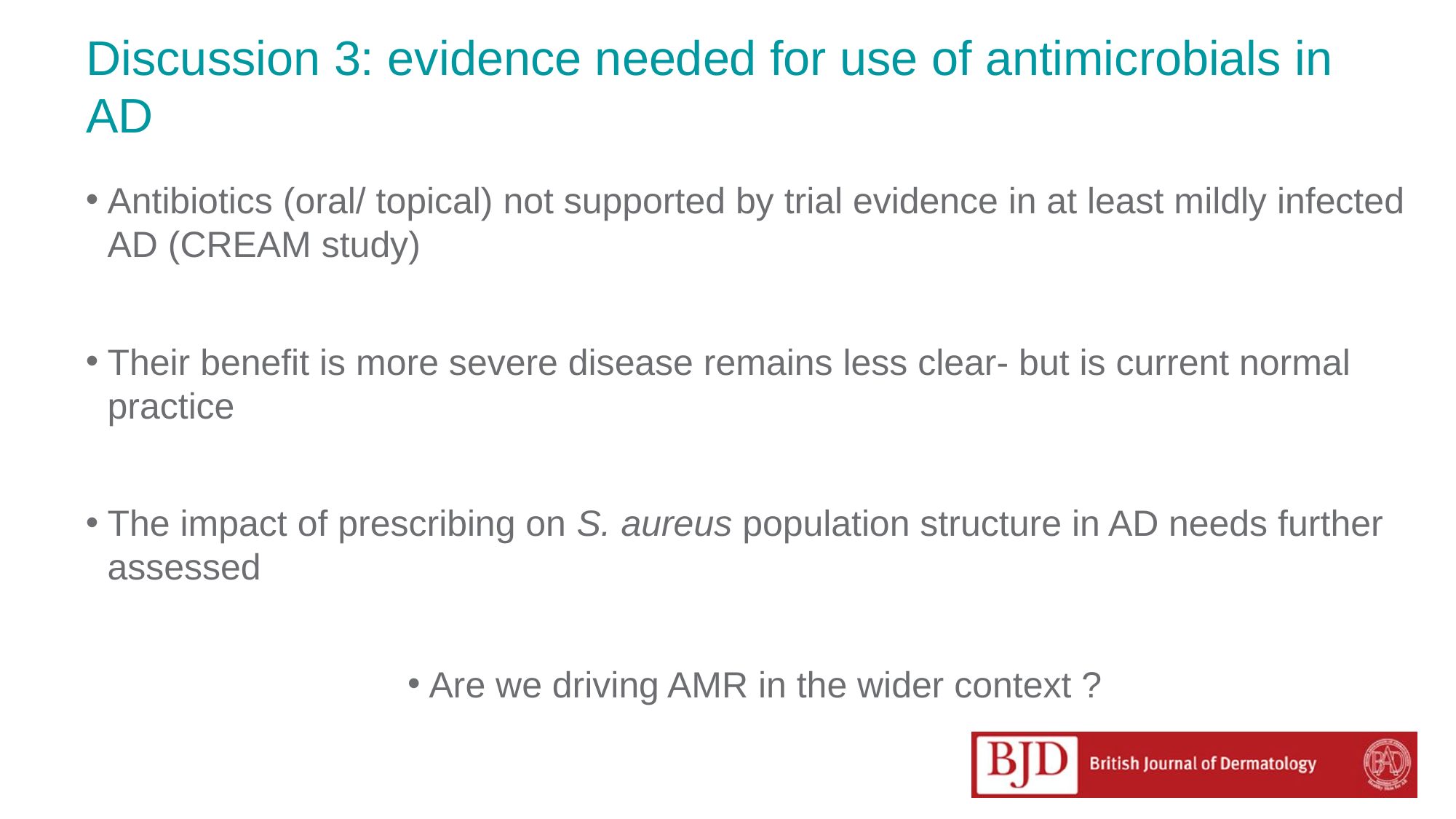

# Discussion 3: evidence needed for use of antimicrobials in AD
Antibiotics (oral/ topical) not supported by trial evidence in at least mildly infected AD (CREAM study)
Their benefit is more severe disease remains less clear- but is current normal practice
The impact of prescribing on S. aureus population structure in AD needs further assessed
Are we driving AMR in the wider context ?

## Slide 14
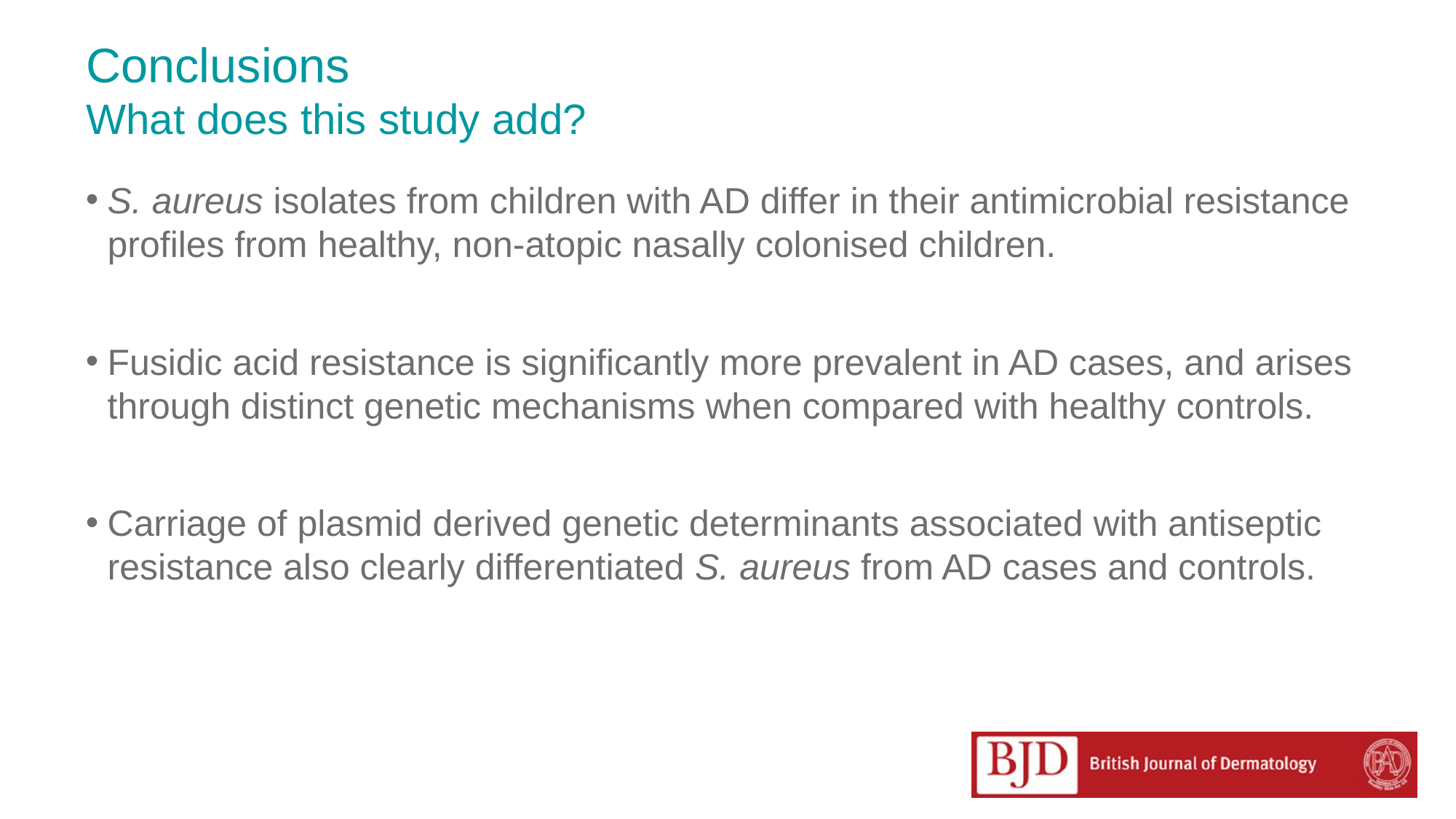

# Conclusions What does this study add?
S. aureus isolates from children with AD differ in their antimicrobial resistance profiles from healthy, non-atopic nasally colonised children.
Fusidic acid resistance is significantly more prevalent in AD cases, and arises through distinct genetic mechanisms when compared with healthy controls.
Carriage of plasmid derived genetic determinants associated with antiseptic resistance also clearly differentiated S. aureus from AD cases and controls.

## Slide 15
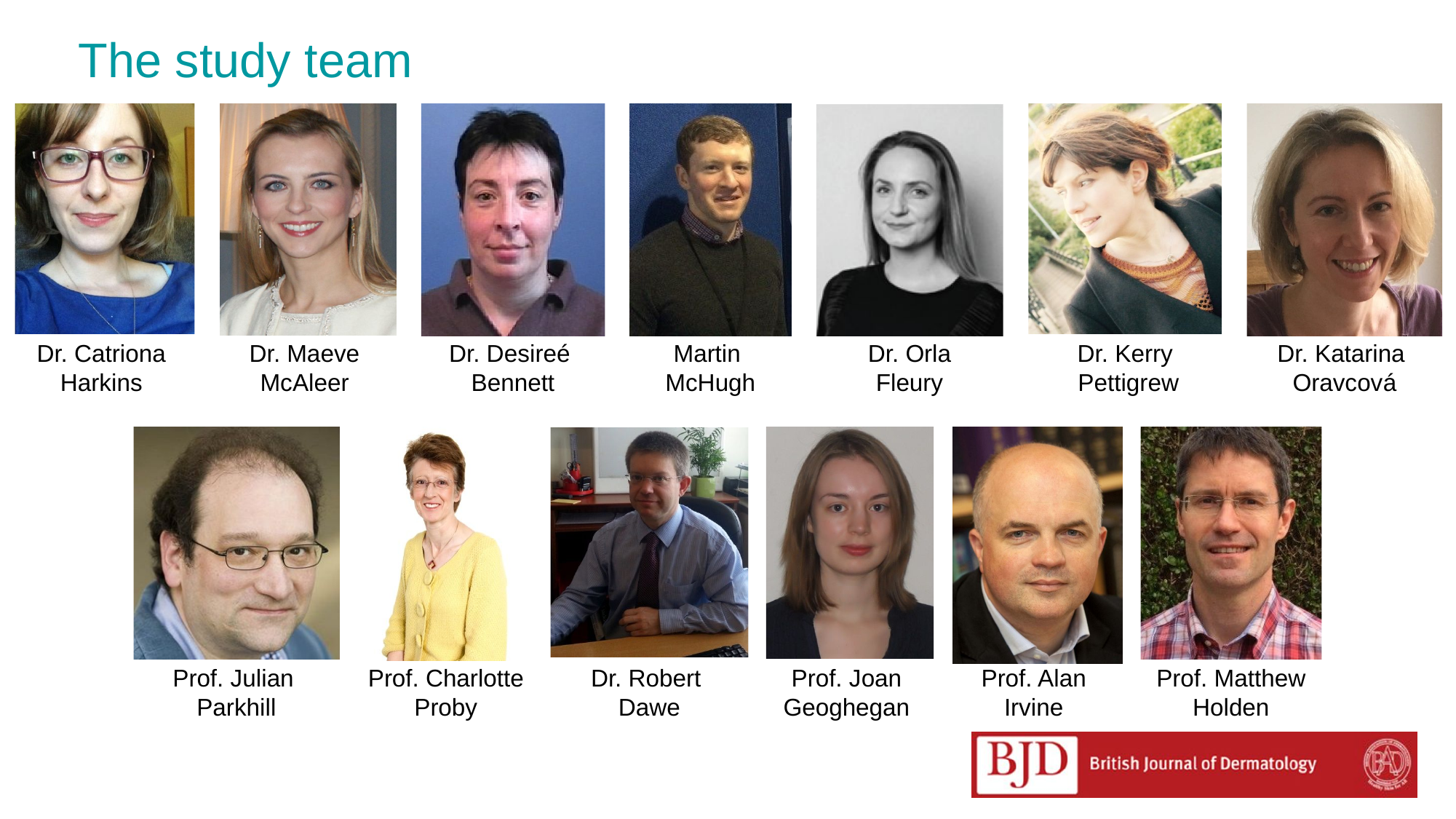

# The study team
Dr. Catriona
Harkins
Dr. Maeve
McAleer
Dr. Desireé
Bennett
Martin
McHugh
Dr. Orla
Fleury
Dr. Kerry
 Pettigrew
Dr. Katarina
Oravcová
Prof. Julian
Parkhill
Prof. Charlotte
Proby
Dr. Robert
Dawe
Prof. Joan
Geoghegan
Prof. Alan
Irvine
Prof. Matthew
Holden

## Slide 16
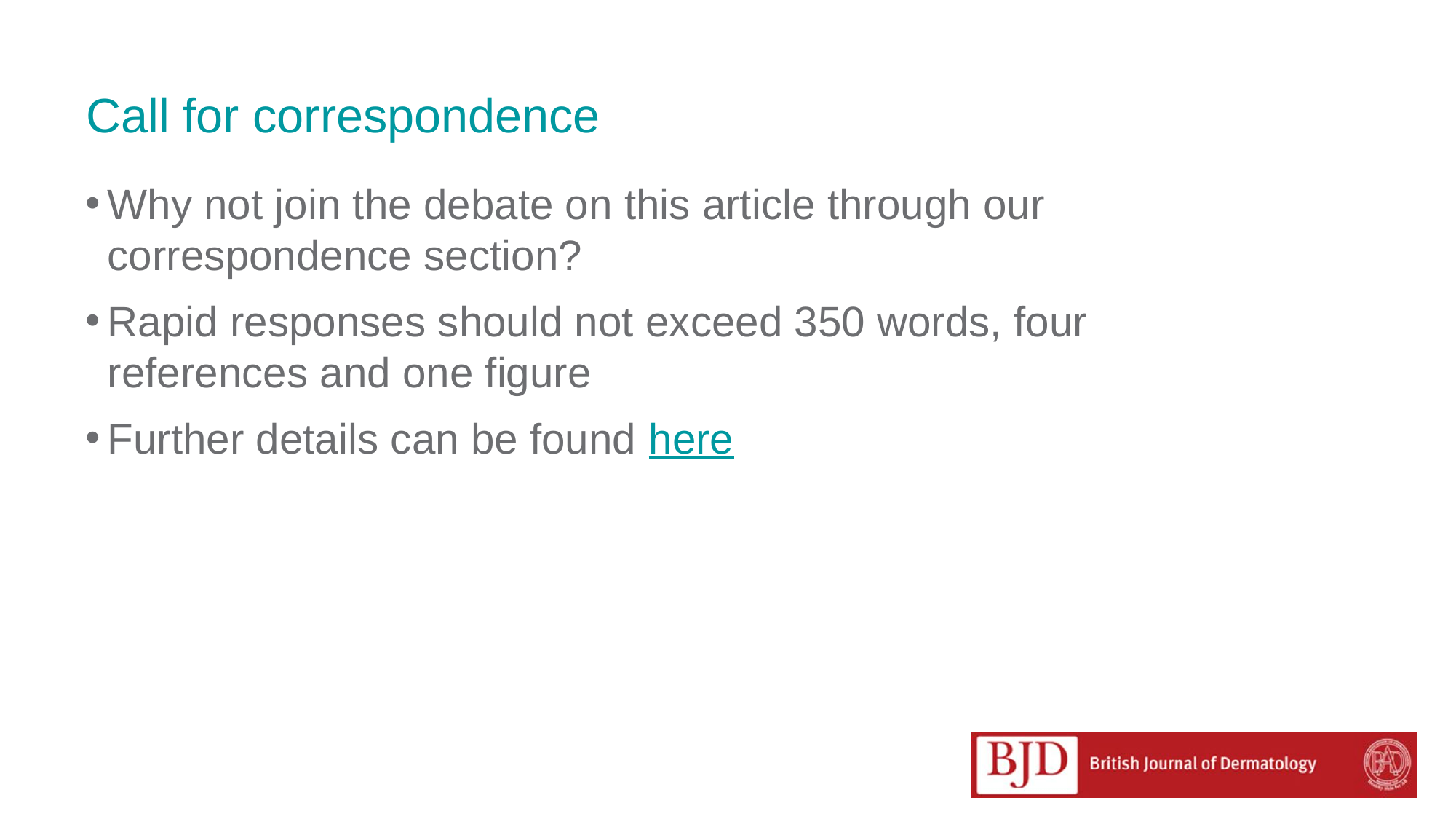

# Call for correspondence
Why not join the debate on this article through our correspondence section?
Rapid responses should not exceed 350 words, four references and one figure
Further details can be found here
